# Supplementary material for: MetaRibo-Seq measures translation in microbiomes
Source: Nat Commun. 2020 Jun 29;11:3268. doi: 10.1038/s41467-020-17081-z (PMC7324362; doi:10.1038/s41467-020-17081-z)
Supplement: Supplementary file 10 — Supplementary Data 7 [file 41467_2020_17081_MOESM10_ESM.zip › File2/Confidence_High_Taxonomy/135997_out.krona.html]

Javascript must be enabled to view this page.

members
magnitude
magnitudeUnassigned
count
unassigned
taxon
rank

135997\_out

9

9
superkingdom
2

phylum
1239
8

186801
class
8

order
186802
8

8

SRS015431\_contig\_number\_80678SRS019068\_contig\_number\_85240SRS020233\_contig\_number\_contig-100\_13621.236412SRS045645\_contig\_number\_6294SRS053356\_contig\_number\_contig-100\_4362.198298SRS056273\_contig\_number\_contig-100\_23238.59939SRS077392\_contig\_number\_36452SRS104693\_contig\_number\_contig-100\_5548.53319
species
1898207

1
1224
phylum

class
28216
1

1
order
80840

995019
family
1

40544
genus
1


SRS054352\_contig\_number\_19864
1
1262976
species
